# Enhanced expression of Cyclin D1 and C-myc, a prognostic factor and possible mechanism for recurrence of papillary thyroid carcinoma

**DOI:** 10.1038/s41598-020-61985-1

**Published:** 2020-03-20

**Authors:** Mojgan Sanjari, Zeinab Kordestani, Moeinadin Safavi, Mahdieh Mashrouteh, Maryam FekriSoofiAbadi, Amirfarhad Ghaseminejad Tafreshi

**Affiliations:** 1 Endocrinology and metabolism Research Center, institute of Basic and clinical physiology Sciences, Kerman University of Medical Sciences, Kerman, Iran; 2 Physiology Research Center, Institute of Basic and Clinical Physiology Sciences, Kerman University of Medical Sciences, Kerman, Iran; 3 PathologyDepartment, Medical Faculty, Tehran University of Medical Science, Tehran, Iran; 4 Modeling in Health Research Center, Institute for Futures Studies in Health, Kerman University of Medical Sciences, Kerman, Iran; 5 Pathology and stem cell research center, Afzalipour Medical School, Kerman University of medical Science, Kerman, Iran; 6 University of British Columbia Vancouver, Vancouver, BC Canada

**Keywords:** Cancer, Endocrinology

## Abstract

A direct association has been shown between Cyclin D1 and C-myc gene expressions and the proliferation of human thyroid tumor cells. Our previous study showed that increased β catenin led to a reduction in disease-free probability in patients with papillary thyroid cancer. This study was designed to investigate Cyclin D1 and C-myc genes as targets for β catenin function in PTC and to determine the association between genes expression and staging, recurrence, metastasis, and disease-free survival of PTC. This study was conducted via a thorough investigation of available data from medical records as well as paraffin blocks of 77 out of 400 patients over a 10-year period. Cyclin D1 and C-myc gene expression levels were measured using real-time polymerase chain reaction (RT-PCR) and the Kaplan-Meier method was used to evaluate disease-free survival. Higher levels of Cyclin D1 and C-myc gene expressions were observed in patients with recurrence by 8.5 (P = 0.004) and 19.5 (p = 0.0001) folds, respectively. A significant positive correlation was found between Cyclin D1 expression and the cumulative dose of radioactive iodine received by patients (r = −0.2, p value = 0.03). The ten-year survival rate in the patients included in this study was 98.25% while disease-free survival was 48.1%. Higher Cyclin D1 and C-myc gene expression levels were observed in patients with recurrence/distant metastasis. Inversely, lower expression of Cyclin D1 and C-myc genes were associated with better survival of patients (SD, 0.142-0.052) (Mantel-Cox test, P = 0.002). The enhancement of Cyclin D1 and C-myc gene expression may be a potential mechanism for recurrence and aggressiveness of PTC.

## Introduction

Thyroid cancer is the most common type of all endocrine-related malignancies^1^. Papillary thyroid carcinoma (PTC) is the most common thyroid cancer, accounting for 80% of all thyroid malignancies^2^. It is extremely important to look into the molecular mechanism of PTC and identify potential biomarkers associated with tumor aggressiveness that may be beneficial for improving treatment. Therefore exploring molecular biomarkers in thyroid cancers has been an area of interest for many researchers^3,4^.

The activation of MAPK and PI3K/Akt pathways, which are normally occurred due to BRAF (more frequently) and RAS family (less frequently) mutations, are two of the most fundamental causes of PTC^5,6^.

The RAS–MAPK pathway controls cell-cycle entry via upregulation of cyclin D1 in several cell types. Eleven  percent cyclin D1 amplification, including 17% of B-RAF V600E melanoma is seen in melanoma, that suggests a potential role of cyclin D1 in intrinsic resistance to B-RAF inhibitors. Upregulation of cyclin D1, a downstream effector of MAPK signaling could be the result of MAPK reactivation. To date, the cyclin D1 amplification in resistant cell lines have not been fully characterized^7^.

Also there are several transcription factors in the Wnt-mediated signaling pathway that are involved in the expression of Wnt/β-cat target genes, such as those encoding C-Myc and Cyclin D1. In this pathway, upon accumulation of β-cat, this protein enters the cell nucleus and interacts with members of the T-cell factor and lymphoid enhancer factor 1 (LEF-1), family of transcription factors. Subsequently, these transcription factors stimulate the expression of Wnt/β-cat target genes, including those encoding C-Myc and Cyclin D1. Hence, transcription of these genes when it is not necessary for cells to proliferate may result in unrestrained growth of cells and tumor development^8^.

Disruption of Wnt cell signaling pathway key regulators i.e.β-catenin and γ-catenin, c-Myc and cyclin D1 is associated with malignant cell transformation. One of the mechanisms linked with protein overexpression is the amplification of Cyclin D1 that was first documented in thyroid cancer. Cyclin D1 is essential in multiple cellular metabolic activities such as mitochondrial activity modulation, cell proliferation and growth regulation, DNA repair, and cell migration control^9^. Previous studies have identified associations between Cyclin D1 expression levels and tumor differentiation, aggressive biological behaviors as well as metastatic disease in thyroid cancer, highlighting the potential role of Cyclin D1 as a prognostic parameter^10–12^.

It seems that frequent alternations in cyclin D1, including amplification, chromosomal translocations, mutations, and activation of the pathways involved in cyclin D1 expression are essential in the development of human cancers, including oral carcinoma^9^.

Cyclin D1 overexpression, one of the markers with greatest potential prognostic value in oral cancer patients, is associated with T and N status, advanced clinical stage, high histological grade, reduced survival, and lack of response to treatment^13^. The expression of the Myc family, that is amplified in the most of hematological neoplasms and solid malignancies, has been shown to enhances tumor growth and drug resistance.C-Myc is the most appearing member of the Myc family that its expression is deregulated in cancers. Deregulation of C-MYC has been detected in thyroid Cancer, especially in PTC^14^.

Moreover, C-myc has also been found to play a fundamental role in malignant transformation. In several tumors including carcinomas of the breast, colon, cervix, small-cell lung cancer, osteosarcomas, glioblastomas and myeloid leukemia, C-myc has been reported to be abnormally overexpressed; making it a potential target for cancer therapy^15,16^.

 Our  previous study has shown that increased β catenin leads to a reduction in disease-free survival. Also  we found a direct relationship between β catenin gene expression and recurrence, TNM (Tumor, Node, Metastasis) stage in PTC^17^.

E-cadherin links to β catenin with two other catenin members (α and γ) at cellular membrane. Perturbation in the expression or function of these complexes results in loss of intercellular adhesion with possible consequent cell transformation and tumor progression. Aberrant expression of β catenin (dispersion in cellular membrane in β catenin/E- cadherin complex or its accumulation in cell nucleus) leads to more relapse and decreases survival rate of papillary cancer survivors^18^.

The aim of this cohort study was to determine if there is an association between Cyclin D1 and C-myc gene expression and the recurrence, metastatic behavior, and disease-free survival. Additionally, in this study we investigated Cyclin D1 and C-myc genes as targets for β catenin function in PTC.

## Materials and Methods

The experimental protocol was approved by the ethical committee of the Kerman University of Medical Sciences, Kerman, Iran (Ethic code No IR.KMU.REC.1394.112). All procedures were performed by the Endocrinology and Metabolism Research Center, Institute of Basic and Clinical Physiology Sciences, University of Medical Sciences, Kerman, Iran (Research code No 940234). All methods used in this study are implemented in accordance with the guidelines and standards set by Declaration of Helsinki.

For this retrospective study, 400 patients diagnosed with PTC were considered. The patients were seen between 2005 and 2015 at a specialist referral clinic in Kerman, a city located in the south-east of Iran. After strict application of the inclusion and exclusion criteria (the details of the inclusion and exclusion criteria have been provided in the appendix), medical records and histopathology slides from 77 patients were reviewed and analyzed. The study was explained to all participants and informed consents were obtained from all enrolled individuals.

From the rest of the subjects which were excluded, 7 had died, 235 has missing or inaccessible surgical records, 58 did not have accessible paraffin blocks and in 23 cases the paraffin blocks were not suitable for RNA extraction. The inclusion and exclusion criteria details can be found in our previously published paper^17^. Thyroid cancer diagnosis was confirmed in all subjects by one pathologist using histopathological slides from paraffin blocks. The AJCC/UICC staging system was employed^19^.

### Study outcomes

The main variables and outcomes assessed in this study include disease-free survival, relapse, and time to recurrence, frequency of surgical procedures, thyroid radioactive iodine uptake, and cumulative radioactive iodine doses. These variables have been specifically defined and explained with details in our previously published study^17^. Summary of definitions are also provided below.

- Relapse: any documented evidence of recurrence that was noted by sonography, isotope scan, PET scan or the development of an elevated serum thyroglobulin (Tg).
- Time to recurrence: the number of months between the time of diagnosis and time of recurrence based on information in the Medical records. Histopathological slides made from paraffin blocks were reviewed to confirm the initial staging in the medical records.
- Disease free survival: defined in 393 patients up to 2015 by information in the medical records or, if the patient did not have a clinic visit that year, by telephone calls.

Additionally, rate of PTC recurrence is calculated and the risk of recurrence and metastasis is reported in the following categories: no metastasis, distant metastasis and lymph node metastasis). The stage of disease is also determined and reported as stages I, II, III and more^19^. Lastly, frequency of receiving radioactive iodine is another variable reported by dividing subjects to groups who have only received a single treatment compared to the ones who were treated multiple times (more than once).

### RNA extraction

The RNA extraction protocol used in this study has been described in a previously published article^17^. A summary of this method has been provided below.

The RNeasy FFPE Kit (Cat. No. 73504, Qiagen Company, Dusseldorf-Germany) was utilized for mRNA extraction. Accordingly, prepared paraffin blocks of tumor tissues with typical macroscopic appearance were used to create 20micron sections (3–5 sections per subject). The sections were then treated with xylene to remove paraffin and extracted mRNA stored at −80 °C.

The RNA concentration of the extracted samples was measured in ng/μl and their optical absorption at A260/A280 nm was measured using Nano Drop (Thermo 2000, USA). Min to max optical absorption of extracted RNA was 1.92–2.03.

Ensuring good quality RNA, the Extracted RNA was loaded on agarose gel. 28srRNA and 18srRNA were detected by Gel Documentation system (Bio-Rad).

Synthesis of first-strand DNA from mRNA was done according to the Thermo Scientific Revert Aid RT Kit (K1621- Thermo Fisher, Darmstadt-Germany). Real-time polymerase chain reaction (RT-PCR) was utilized to evaluate the relative expression of CyclinD1 and C-myc genes using Real Q Plus 2x Master Mix Green with high ROX (Ampliqon Company, Denmark) and primers designed by primer 3 software. The sequences of the CyclinD1 PCR primers were forward primer, 5′-GCG GAG GAG AAC AAA CAG-3′; reverse primer, 5′-GCG GTA GTA GGA CAG GAA-3′, C-myc forward primer, 5′-GCGACTCTGAGGAGGAA-3′; reverse primer, 5′-TGCGTAGTTGTGCTGATG-3′ and β actin (house housekeeping) forward primer, 5′-ACC ACC TTC AAC TCC ATC ATG-3′; revers primer, 5′-CTC CTT CTG CAT CCT GTC G-3′.

The details of the RT-PCR protocol used have been provided in the appendix.

The PCR cycle at which the fluorescent signal of the reporter dye crosses an arbitrarily placed threshold, which is quantitative endpoint for RT-PCR, is referred to as the threshold cycle or CT (13). The Δ CT formula (Δ CT = CT target gene – CT housekeeping gene) was used to normalize the expression of CyclinD1 and C-myc (target genes) against β actin (housekeeping gene). Given the inverse relationship between gene expression and Δ CT, smaller Δ CT values indicate higher gene expression^20,21^. Fold change or a fold-difference of expression levels in two groups = 2^−ΔCT^/2^−ΔCT^ ^21^.

### Immunohistochemistry technique for cadherin

All Paraffin blocks were cut at 3–4 µm sections and were dewaxed in xylene at 37 °C. A 0.5% hydrogen peroxide in methanol was used for blocking endogenous peroxide activity. Subsequently, the sections were placed in a steamer filled by EDTA buffer (pH = 8) and the heat-mediated antigen retrieval was fulfilled. Sections were then incubated with E-cadherin monoclonal antibody. Finally,  envision secondary antibody was utilized to react with 3,3-diaminobenzidine (DAB) to visualize immunostaining.

### Statistical analysis

Variables are primarily expressed with descriptive scales. Nonparametric tests are applied to compare the expression levels of the CyclinD1 and C-myc genes between groups. Kaplan-Meier and Cox regression are utilized for survival analysis. Statistical analyses are carried out using SPSS 22. Shapiro-Wilk test is used to assess the distribution of CyclinD1 and C-myc gene expression data which shows to be non-significant and normally distributed (CyclinD1 p = 0.2 C-myc p = 0.3). Descriptive characteristics are summarized in Table 1.

**Table 1:** Descriptive Analysis of Patient Characteristics.

Variables	result
Age^a^ (year)	38.7 ± 14.1
Sex (female)^b^	81.8
Expression^a,c^	3.54 ± 4.77
Cyclin D1	
C-myc	4.74 ± 4.84
Stages^b^	
I	70.3
II	12.5
≤III	17.2
Recurrence (Yes)^b^	53.2
Metastasis^b^	
None	74.2
Lymphatic	16.7
Distance	9.1
Radioactive Iodine treatment^b^	
One time	58
Two times	18.8
Three times	14.5
Four times	8.7
Radioactive Iodine dosage^a^ (mCi)*	241.73 ± 178.89
Follow up time(months)^a^	102.5 ± 40.4

^a^Mean ± SD.

^b^Percent.

^c^Cycle threshold (ΔCT) values are presented in this table to represent Cyclin D1,C-myc expression levels in tumors.

ΔCT = Cyclin D1, C-myc CT – β-actin CT.

## Results

This retrospective study was performed on collected data from 77 subjects who had been diagnosed with papillary thyroid carcinoma during the past 10 years. The ten-year survival rate in these patients was 98.25% and disease-free survival was 48.1. There was no statistically significant correlation between the age of patients and CyclinD1 and C-myc expression (r = 0.1, P value = 0.3).

The relationships between CyclinD1 and C-myc gene expression and the patients’ gender, recurrence status, number of radioactive iodine treatments, metastases, and tumor stage are shown in Table 2.

**Table 2:** Association of CyclinD1 and C-myc gene expression with other variables.

Variables	CyclinD1 expression^*,a^	Fold change^b^	P value	C-myc expression^*,a^	Fold change^b^	P value
Sex*						
Male	0.175 ± 0.66		0.2	0.169 ± 0.66		0.5
Female	0.222 ± 0.833			0.2 ± 0.833		
Recurrence*						
Yes	0.5 ± 0.256	8.5 (yes vs. no)	0.004	0.37 ± 0.27	19.5 (yes vs. no)	<0.0001
No	0.196 ± 0.196			0.14 ± 0.2		
Stage*						
I	0.25 ± 0.2	2.5 (2 vs. 1)	0.7	0.188 ± 0.21	3 (2 vs. 1)	0.5
II	0.37 ± 0.166	0.2 (3 vs. 2)		0.263 ± 0.151	0.2 (3 vs. 2)	
≤III	0.217 ± 0.227	0.6 (3 vs. 1)		0.163 ± 0.243	0.5 (3 vs. 1)	
Radioactive Iodine treatment^*^						
One time(1)	0.212 ± 0.227	5.2 (2 vs. 1)	0.03	0.188 ± 0.27	6.3 (2 vs. 1)	0.1
Two time(2)	0.434 ± 0.243	0.3 (3 vs. 2)		0.384 ± 0.2	0.3 (3 vs. 2)	
Three time(3)	0.263 ± 0.172	27 (4 vs. 3)		0.238 ± 0.14	3.2 (4 vs. 3)	
Four time(4)	−0.999 ± 0.322	52 (4 vs. 1)		0.4 ± 0.277	6.5 (4 vs. 1)	
Radioactive Iodine dosage**	r = −0.4		<0.0001	r = −0.4		<0.0001
Metastasis*						
None	0.25 ± 0.227	24.2 (d vs. n)	0.05	0.181 ± 0.238	6 (d vs. n)	0.1
Lymphatic(l)	0.33 ± 0.175	2 (l vs. n)		0.243 ± 0.25	3.5 (l vs. n)	
Distance(d)	−1.66 ± 4.2			0.345 ± 0.188		
C-myc Gene Expression**	r = 0.7		<0.0001			

^*^(mean ± SD).

** Correlation covariate.

^a^1/ΔCT values are presented in this table to represent CyclinD1 and C-myc expression levels in tumors.

Δ Ct = target gene (Cyclin D1, C-myc) Ct - housekeeping gene (beta-actin) C t.

^b^fold change = 2^−ΔCT^ CyclinD1,Cmyc  /2^−ΔCT^ beta -actin.

P Value is significant at 0.05 levels.

CyclinD1 and C-myc gene expression were similar in males and females. In patients with recurrence, it was shown that CyclinD1 expression was higher and it was statistically significant (8.5 fold increase) P = 0.004 and C-myc expression was higher and it was statistically significant (19.5 fold increase, P = 0.0001).

Patients with distant metastasis had higher CyclinD1 and C-myc gene expression than patients without distant metastasis. These differences are statistically significant (24.2 fold increase) for CyclinD1 (P = 0.05) but not for C-myc gene expression (P = 0.1).

Patients with stage II had a higher Cyclin D1 and C-myc gene expression compared to patients with stage I, though this observed difference is not statistically significant (CyclinD1 P = 0.7) (C- myc P = 0.5). Moreover, patients who received at least three or four radioactive iodine treatments had significantly higher CyclinD1 gene expression by 52 folds (P = 0.03) compared to patients who only received a single radioactive iodine treatment.

There was a reverse correlation between dosage of radioactive iodine with Cyclin D1 and C-myc gene expression. It means that higher radioactive iodine was used in patients with lower gene expression.Due to a 52-fold increase in the expression of cyclin D1 in patients having the most radioactive iodine intake (more than 4 times), this oncogene is associated with aggressive disease. By reassessing and ensuring r = −0.4, we found that the decrease in cyclin D1 gene expression in patients receiving high doses of radioactive iodine(mCi) may be an indication of the therapeutic effect of radioactive iodine in these patients, although its mechanism is unclear. However, due to limitations in sample size, we were not able to classify individuals as subgroups by dose of radioactive iodine intake and measure gene expression levels in these individuals (Table 2).

**Figure 1:**
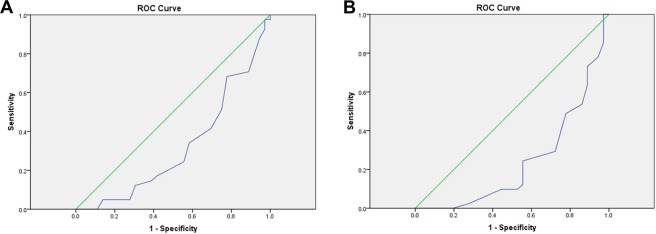
(**A**) ROC curve of CyclinD1 gene expression for recurrence diagnosis. (**B**) ROC curve of C-myc gene expression for recurrence diagnosis. We designed an ROC curve to determine a cutoff point for Cyclin D1 and C-myc gene expression as a way to predict the probability of recurrence. Area under the ROC curve (AUC) was 32% for CyclinD1 (**A**) and 24% for C-myc (**B**), which were both below the 50% standard reference line (the reference line is colored in green). Given that the test accuracy is below the 50% cut off point for expression of both genes, recurrence probability could not be adequately and reliably evaluated (**A**,**B**).

We designed an ROC curve to determine a cutoff point for Cyclin D1 and C-myc gene expression as a way to predict the probability of recurrence. Area under the ROC curve (AUC) was 32% for CyclinD1 (Fig. 1A) and 24% for C-myc (Fig. 1B), which were both below the 50% standard reference line. Given that the test accuracy is below the 50% cut off point for expression of both genes, recurrence probability could not be adequately and reliably evaluated (Fig. 1A,B).

**Figure 2:**
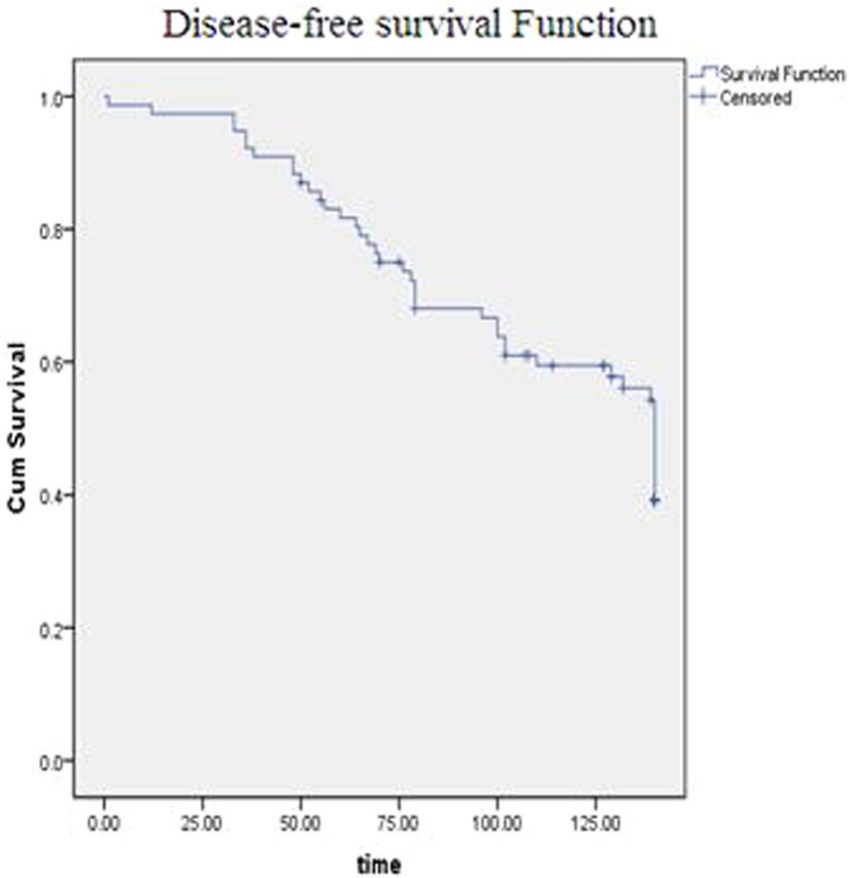
Disease-free survival plot for recurrence of PTC. Kaplan-Meier method was applied in order to determine disease-free survival of patients as recurrence of their disease. Mean and median of the time difference between baseline and follow up were between 108.8 (95% CI: 99.5–118.2) and 140 (95% CI: 134.1–145.8) months, respectively.

Kaplan-Meier method was applied in order to determine disease-free survival of patients as recurrence of their disease. Figure 2 demonstrates the cases with reported survival and disease recurrence. Mean and median of the time difference between baseline and follow up were between 108.8 (95% CI: 99.5–118.2) and 140 (95% CI: 134.1–145.8) months, respectively.

Cyclin D1 and C-myc gene expressions are categorized based on corresponding percentiles (Table 3).

**Table 3:** Characteristics of the categorized CyclinD1, C-myc gene expression.

Gene	Category	number	Percentile	Min – Max*	Mean ± SD*	Mean of follow up time(months)	95% CI of follow up time(months)
CyclinD1	1	29	33.3	0.1–1	−1 ± 0.5	91.6	76.1–107.2
	2	26	66.6	0.2–0.5	0.3 ± 0.9	110.4	93.0–127.8
	3	22	99.9	0.05–0.1	0.1 ± 0.3	128.7	117.3–140.1
C- myc	1	29	33.3	0.1–0.3	−0.03 ± 0.3	93.0	77.0–109.1
	2	23	66.6	0.1–0.2	0.2 ± 1.2	105.7	89.2–122.2
	3	25	99.9	0.06–0.1	0.1 ± 0.3	129.7	116.2–143.3

^*^1/ΔCT values are presented in this table.

**Figure 3:**
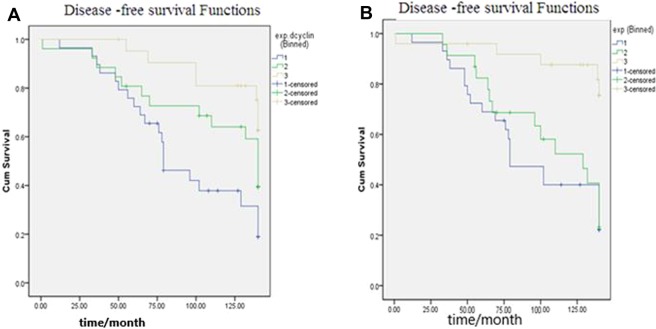
(**A**) Disease-free survival plot for recurrence of PTC based on Cyclin D1 gene expression. (**B**) Disease-free survival plot for recurrence of PTC based on C-myc gene expression. We found that better survival of patients (lower recurrence) was associated with lower Cyclin D1 (Mantel-cox test, P = 0.003) (**A**) as well as lower C-myc gene expression (Mantel-cox test, P = 0.001) (**B**).

We found that better survival of patients (lower recurrence) was associated with lower Cyclin D1 (Mantel-cox test, P = 0.003) Fig. 3. A. as well as lower C-myc gene expression (Mantel-cox test, P = 0.001) Fig. 3B.

We performed a multivariate survival analysis (cox regression analysis) for evaluation of confounders and found the optimal model fitted to the data. Age, sex, CyclinD1 and C-myc gene expression, stage of the disease and radioactive iodine dosage were considered as confounders. Finally, we found that iodine dosage and C-myc gene expression were the confounding variables in survival (in this study recurrence was defined as the end event) and remained in our model. Table 4 shows the details of this analysis.

**Table 4:** Multivariate survival analysis (Cox regression analysis) for determinants of recurrence.

variables	coefficient	P value	Odds ratio (OR)	95% CI for OR	
C-myc Gene expression	−0.1	0.007	0.89	0.83	0.97
Iodine dosage	0.003	0.001	1.003	1.002	1.005

Table 4 demonstrates that for every unit number increase in C-myc gene expression, log of the relative risk of recurrence increases by 0.1. It further shows that there is a direct correlation between the C-myc gene expression and log of the relative risk of recurrence. This observation with the odds ratio of 0.89 (CI: 0.83–0.97) that is statistically significant for C-myc gene expression confirms its aggressive role in disease recurrence. However, Cyclin D1 gene expression does not show to have an effect on increasing the relative risk of recurrence.

Also, in this table we can see that an increased unit number in the Iodine dosage causes a 0.003 increase in log of the relative risk of recurrence, but OR and its CI show that there is no statistically significant difference between them. OR = 1.003 showed that it is a weak risk factor for statistical significance.

A Pearson test was performed to detect potential correlations between gene expressions (Beta catenin, C-myc, cycline D1). As it is shown in Table 5, beta catenin gene expression is significantly correlated with C-myc and cycline D1 gene expressions, with the same correlation coefficient (r = 0.5, P < 0.0001).

**Table 5:** Correlation between C-myc, Cyclin D and β catenin gene expression.

Correlations				
		C-myc	CyclinD1	B catenin
C-myc	Pearson Correlation	1	0.763**	0.502**
N = 77	P	—	0.001	0.001
cyclin D1	Pearson Correlation	0.763**	1	0.501**
N = 77	P	0.001	—	0.001
Β catenin	Pearson Correlation	0.502**	0.501**	1
N = 77	P	0.001	0.001	—

**Correlation is significant at the P ≤ 0.01 level (2-tailed).

We reported the hazard rates of recurrence for each year of follow up in Table 6. Overall hazard rate of recurrence up to end of the study (140 months) was 0.005.

**Table 6:** Hazard rates of recurrence for each year of follow up.

Interval start time	Hazard ratio	Standard error of Hazard ratio
0	0.0	0.0
12	0.0	0.0
24	0.0	0.0
36	0.0	0.0
48	0.01	0.0
60	0.01	0.0
72	0.01	0.0
84	0.0	0.0
96	0.01	0.0
108	0.0	0.0
120	0.0	0.0
132	0.05	0.02

## Discussion

Advances in genomics, proteomics, and molecular pathology have increased the possibility of the measurement of biomarkers with clinical significance in thyroid cancer^22^.

We have previously shown that β catenin gene expression is positively correlated with recurrence, distant metastasis and TNM, stage in PTC (the same population)^17^.

The nuclear coactivator β catenin associated with T-cell factor and lymphoid enhancer factor (LEF) induces gene transcription such as C-myc and Cyclin D1. Transcription of target genes when proliferation is not required leads to the unrestrained growth of cells^23^.

Therefore, we designed this study to investigate the possible mechanism for βcatenin function in recurrence and aggressiveness of the PTC. We also tried to answer whether c-myc and CyclinD1 are relevant target genes for β catenin levels of patients with PTC.

The results of the present study illustrate a direct association between both Cyclin D1 and C-myc mRNA levels and the recurrence of PTC. Additionally, patients with frequent recurrence showed higher levels of Cyclin D1and C-myc mRNA expression compared to those without recurrence. Moreover, a direct correlation was observed between the relative expression of Cyclin D1 and C-myc gene and radioiodine dosage. It was also seen that with progress in the stages of the disease, expression of Cyclin D1 and C-myc genes were increased. Based on our study, Cyclin D expression was significantly higher in patients with metastasis.

There was no significant increase in C-myc expression in patient with metastatic disease.

Previous studies have also shown that overexpression of beta catenin, CyclinD1 and C-myc play pivotal roles in thyroid cancer^17,24,25^.

In a study carried out by Sakr *et al*.^25^, they were able to illustrate the exclusive nuclear expression of C-myc in thyroid carcinoma and nodular hyperplasia using tissue microarrays generated from follicular cell-derived thyroid carcinomas. In cases of nodular hyperplasia and well-differentiated carcinomas, C-myc expression was found to be weakly positive. In addition, they were able to detect a direct correlation between nuclear overexpression of C-myc and tumorigenesis/dedifferentiation in follicular cell derived thyroid carcinomas^25^.

Cyclin D1 overexpression was also found in papillary micro carcinomas that was strongly associated with increased tumor aggressiveness, lymph node metastases, and cell proliferation^11^. Despite the observed association between Cyclin D1 overexpression and lymph node metastases in various malignancies, the expression of cyclin D1 in PTC may not depend on the amplification of this gene^26^. Our suggestion  in this case is that the expression of Cyclin D1 in PTC may not depend on the amplification of this gene but this over expression due to on alterations in b-catenin signaling^11,26^.

Similarly to our study, Perna *et al*. reported increased cyclin D1 in many tumors such as PTC. Curcumin ameliorated the upregulation of cyclin D1 which was occurred as a result of the upregulation of β catenin in WNT signaling pathway^12^.

In accordance with previous research and based on the results of this study, we found that the expression of Cyclin D1 and C-myc genes were positively correlated with disease recurrence, similar to changes in β catenin gene expression. Hence, it seems that β catenin function in PTC patients is a result of induced Cyclin D1 and C-myc genes.

On the other hand, β catenin is closely linked to E-cadherin which plays an important role in cellular connections and cell adhesion^27^. Decreased expression of β catenin and E-cadherin in the cell membrane is associated with the loss of cell adhesion and cohesion, which may result in development of invasive carcinoma and tumors. This allows the cells to be isolated from the primary site and attack their surrounding tissue, causing lymph node and even distant organs metastasis^18,28^. For this reason, immunohistochemically (IHC) localization of E-cadherin and its relationship to survival was carried out in these samples. Unfortunately, the amount of remaining paraffin blocks (sample size) was too little to perform viable statistical analyses.We recommend additional studies with larger sample sizes to determine the relationship between E-cadherin and survival in patients with PTC.

The limitations of this retrospective cohort study were the difficulty in accessing patient information, either from follow-up contact or medical records. Secondly, RNA quality of some paraffin blocks may have been affected due to long term storage. These factors excluded many patients from the study, from a sample size of 400 to 77. Due to the retrospective design of the study access to pre-operative information was not possible and the analysis was based on stage I, II, III etc. The cellular localization of cyclin D1 is an important parameter in the role play of this factor which should have been tracked by immunohistochemistry in the present study. However, immunohistochemically (IHC) localization of E-cadherin and its relationship to survival was carried out in the samples. Unfortunately, the amount of remaining paraffin blocks (sample size) was too small to perform viable statistical analyses.

Additionally, the gain of function and loss of function approaches could be used to confirm the results.

Our results demonstrate that enhancement of β catenin expression and induction of the C-myc (target gene) leads to aggressiveness and recurrence in patient with papillary thyroid carcinoma. It seems that inhibition of this pathway may be useful in developing more targeted therapies. We aim to attempt this approach in the future.

## Conclusion

Our findings suggest that induction of C-myc and Cyclin D1 gene transcription is a possible pathway for beta catenin, as an underlying source of papillary thyroid cancer’s aggressiveness.
